# Can the Japanese National Clinical Database risk calculator predict long-term survival of patients who undergo palliative segmentectomy for primary lung cancer?

**DOI:** 10.1007/s11748-021-01585-6

**Published:** 2021-01-28

**Authors:** Tomoyuki Nakano, Hiroyoshi Tsubochi, Mitsuru Maki, Kentaro Minegishi, Tomoki Shibano, Yoshihiko Kanai, Shinichi Otani, Shinichi Yamamoto, Kenji Tetsuka, Shunsuke Endo

**Affiliations:** 1 Department of General Thoracic Surgery, Jichi Medical University, 3311-1 Yakushiji, Shimotsuke, Tochigi 329-0498 Japan; 2 Department of General Thoracic Surgery, Jichi Medical University Saitama Medical Center, Saitama, Saitama Japan

**Keywords:** Palliative surgery, Risk calculator, Pulmonary segmentectomy

## Abstract

**Objectives:**

Selection criteria for palliative limited surgery in patients with non-small cell lung cancer (NSCLC) can vary by institution or surgeon. We retrospectively reviewed outcomes of poor-risk patients who underwent palliative segmentectomy (PS), using the National Clinical Database Risk Calculator (RC).

**Methods:**

We retrospectively analyzed medical records of patients with NSCLC tumors ≥ 20 mm and consolidation/tumor ratios ≥ 0.5 on computed tomography, who underwent PS from January 2009 to March 2016. Median follow-up time was 47 months (range 2–102 months).

**Results:**

We enrolled 67 patients (median age: 73.0 years), of whom 54 received thoracoscopic surgery and 28 received medial lymph-node dissection. The RC’s mean predictive probability rate for perioperative mortality or severe complications was 7.1%. Of the 67 patients, 24 patients (43.0%) suffered post-surgical complications, including 2 (3%) who died in hospital; 17 eventually suffered NSCLC recurrences and/or metastases, 11 eventually died from NSCLC, and 17 died from other diseases. Five-year overall survival (OS) was 59.4%. When the patients were divided into high-risk (HR) and low-risk (LR) groups based on the RC, 5-year OS was significantly less in the HR group (43.9%) than in the LR group (82.2%; *P* < 0.05).

**Conclusion:**

The RC, which was developed primarily to determine perioperative risk, can predict long-term prognosis for compromised patients who undergo PS.

**Supplementary Information:**

The online version contains supplementary material available at 10.1007/s11748-021-01585-6.

## Introduction

Lobectomy is a recommended standard surgical procedure for non-small cell lung cancer (NSCLC), based on results of a randomized-controlled trial by the Lung Cancer Study Group (LCSG) that compared sublobar resection (SLR) with lobectomy [1]. SLR has been conventionally used as a compromise procedure for poor-risk patients with lung cancer when lobectomy is not considered feasible [2]. However, selection criteria for limited palliative surgery for NSCLC are not standardized, and can differ among institutions and surgeons. Furthermore, SLR’s safety and prognostic effect on long-term survival compared with lobectomy, and patient subgroups that can potentially benefit from SLR are unclear [3]. However, our institutional preference is to perform pulmonary segmentectomy for wider surgical margins and more detailed nodal assessments.

This study investigated long-term outcomes of palliative segmentectomy (PS) for NSCLC, and the prognostic factors for poor-risk patients who undergo limited surgery. We used the risk calculator (RC) from the Japanese National Clinical Database (NCD) to assess poor-risk patients who received PS.

## Methods

### NCD and risk calculator

The Japanese NCD adopted a “web-based collection” in 2011 with significant support from Japan Surgical Society [4]. The NCD is a nationwide collaboration associated with the Japanese Surgical Board Certification System, in which data on 1.6 million surgical procedures from > 4000 hospitals were collected in 2014. It is linked to the second level in the specialty chest surgery hierarchy through a web-based conversion, both of which are supported by the Japanese Board of General Thoracic Surgery [5].

The RC indicates the predictive incidence ratio of surgery-related death and major complications, based on a model of lung cancer surgery risk derived from the Japanese nationwide web-based database of 78,594 patients during 2014–2015 [6]. In this study, the primary outcome measures were surgical mortality, and the combined outcome of mortality and major morbidity. Operative mortality included patients who died within 30 days after surgery, and major morbidity was defined in accordance with the Society of Thoracic Surgeons (STS) risk models [7, 8]. Endo et al. [6] reported that the most common cause of major morbidity was respiratory failure after pneumonia and atrial arrhythmia. Multivariate risk models were developed in the report [6], and the final logistic model with odds ratios (ORs) and 95% confidence intervals (CIs) is presented in Supplementary Table 1, which shows associations between mortality or mortality/major morbidity, and patient baseline characteristics. Nineteen variables were associated with mortality, and 25 variables were associated with mortality/major morbidity. The RC’s mean predictive probability of perioperative mortality or major morbidity (PPMM) can be calculated based on OR of each variable in above risk model list (Supplementary Table 1). If we access Internet website (https://registry3.ncd.or.jp/karte/page/feedback/riskcalc?specialist_id=A00056_001) online, and enter 20 variables associated with mortality/major morbidity (sex, age, performance status, pulmonary function tests, preoperative comorbidity, smoking history, induction therapy, radiological tumor size, clinical stage, surgical procedure, histology, etc.), it will produce a predictive incidence ratio of surgery-related death and severe complications.

### Patients

This retrospective study was approved by the Ethics Committee at Jichi Medical University. Between January 2009 and March 2016, 2241 patients underwent pulmonary resection for NSCLC at two related institutions, including 282 patients who underwent pulmonary segmentectomies. Among these 282 patients, we obtained data from medical records for patients who underwent PS during this period, because they were considered poor risks for more invasive procedures. We included patients with NSCLC who received PS, and whose tumors were more than 20 mm and consolidation/tumor (C/T) ratios on computed tomography (CT) were more than 0.5 [9, 10]. Their clinicopathological staging was determined according to *General Rule for Clinical and Pathological Record of Lung Cancer* (7th edition) by the Japanese Lung Cancer Society [11]. Seven patients underwent segmentectomies due to incomplete resections (R2) were excluded in this study. Four patients had a preoperative diagnosis of distant metastasis, intraoperative pleural dissemination was revealed in two patients, and intraoperative malignant pleural effusion was revealed in one patient.

Preoperative evaluation consisted of physical examination, past medical history, social history, pulmonary function tests, chest and abdominal CT, brain magnetic resonance imaging (MRI), and positron emission tomography (PET). Reasons for limited surgery were poor pulmonary function, various comorbidities, metachronous or simultaneous multiple pulmonary lesions, or advanced age (more than 80 years).

Patients were followed up after surgery at 3- to 6-month intervals, with physical examination, chest radiography, blood tests that included tumor marker levels, chest and abdominal CT, brain MRI, and PET if necessary. Local recurrence was defined as a tumor occurring at the staple line, ipsilateral hilar or mediastinal lymph nodes, or pleural cavity, including the ipsilateral lung parenchyma.

### Surgical technique

We performed thoracoscopic pulmonary resections using a five-port non-rib-spreading technique with a 45° thoracoscope through a 10.5-mm trocar and four ports protected with a 5.0- or 10.5-mm trocar. The skin incision of one port was extended according to the size of the specimen. Pulmonary resection was performed through the thoracotomy using a 20- to 30-cm posterolateral skin incision, splitting the anterior serratus muscle, dorsal latissimus muscle, and rib. The fourth, fifth, or sixth inter costal space was used. The segmental bronchi and vascular were closed with a stapler, and the minor vascular branches and small bronchi were ligated with sutures. A stapler, and electric or ultrasonic cauterization were used to divide the intersegmental plane according to the preserved bronchi and the intersegmental pulmonary vein. The parenchymal surgical margins were at least 2 cm where possible. The pathologic findings showed that surgical stump was negative in all patients. We defined left upper, left lingular, S6, and basal segmentectomies as simple segmentectomies. Lymph-node dissection was selected from systematic dissection or sampling the hilar and mediastinal nodes, based on the patient’s clinical status and tumor clinical stage.

### Statistical analysis

Differences were statistically evaluated using Student’s *t* test for numerical variables and Chi-square test for categorical variables. *P* < 0.05 was considered significant. Overall survival (OS) and Recurrence-free survival (RFS) curves were generated via the Kaplan–Meier method. Statistical differences between groups were evaluated by the log-rank test. Receiver-operating characteristic (ROC) curve of the RC’s PPMM for predicting overall survival was generated to determine cut-off value that yielded optimal sensitivity and specificity. Univariate and multivariate analyses using a logistic regression model were also performed to evaluate the significance of factors related to recurrence. All statistical analyses were performed using the StatMate V software package (ATMS Co., Ltd., Tokyo, Japan) and EZR (Saitama Medical Center, Jichi Medical University, Saitama, Japan). We consulted Japan Institute of Statistical Technology (Tokyo, Japan) for methods of analysis.

## Results

### Clinicopathological features

Characteristics and clinicopathological features of all patients are shown in Table 1. Enrolled patients included 56 men (83.6%) and 11 women (16.4%), whose mean age was 73.0 years (range 50–90 years). The RC’s mean PPMM for the 67 patients was 7.1%. Thoracoscopic surgery was performed for 54 patients (80.6%), and medial LN dissection was performed for 28 patients (41.8%). The mean postoperative hospital stay was 20.4 days. Twenty-nine patients (43.3%) had complications after surgery; 2 patients (3.0%) died in hospital.

**Table 1:** Characteristics of the study population

	*N*	%
Age, years, mean (range)	73.0 (50–90)	
Sex		
Male	56	83.6
Female	11	16.4
Performance status (PS)		
PS0	56	83.6
PS1	11	16.4
Brinkman index, mean (range)	1194.5 (0–5000)	
%VC, %, mean (range)	102.4 (51.5–148.4)	
FEV_1_%, %, mean (range)	66.8 (34.2–99.3)	
Interstitial pneumonia	15	22.4
Chronic obstructive pulmonary disease	42	62.7
Diabetes mellitus	12	17.9
Ischemic heart disease	8	11.9
Central nerve systemic disorder	9	13.4
Other neoplasm	19	28.4
Tumor size on CT, mm, mean (range)		
Maximum	35.9 (21–93)	
Solid component	28.4 (16–93)	
Consolidation/tumor ratio, mean (range)	0.79 (0.51–1.00)	
SUV of PET-CT, mean (range)	8.7 (0.99–28.0) ^a^	
Clinical stage		
IA	21	31.3
IB	32	47.8
IIA	3	4.5
IIB	4	6.0
IIIA	7	10.4
Location of tumor		
Right upper lobe	10	15.0
Left upper lobe	25	37.3
Right lower lobe	25	37.3
Left lower lobe	7	10.4
Predictive probability with NCD RC, %, mean (range)	1.0 (0–7.3)	
Mortality (when undergoing lobectomy)	2.3 (0.1–19.4)	
Mortality or major morbidity (when undergoing segmentectomy)	7.1 (1.4–22.1)	
Mortality or major morbidity ((when undergoing lobectomy)	10.0 (2.0–29.5)	
Major reason for limited surgery		
Poor pulmonary function and respiratory disease	44	65.7
Comorbidities	9	13.4
Multiple pulmonary lesions	8	11.9
Age (≥ 80 years)	4	6.0
Others	2	3.0
Surgical approach and procedure		
Thoracotomy	13	19.4
Thoracoscopic surgery	54	80.6
Simple segmentectomy	48	71.6
Complex segmentectomy	19	28.4
Nodal dissection (ND)		
ND0 or ND1	39	58.2
ND2	28	41.8
Pathologic tumor size, mm, mean (range)	32.6 (13–95)	
Histological type		
Adenocarcinoma	33	49.3
Squamous cell carcinoma	23	34.3
Others	11	16.4
Visceral pleural invasion		
pl(−)	41	61.2
pl( +)	26	38.8
Lymphatic vessel invasion		
ly(−)	41	77.4
ly( +)	12	22.6
Blood vessel invasion		
v(−)	24	48.0
v( +)	26	52.0
Pathologic lymph-node involvement		
N0	59	88.1
N1 or N2	8	11.9
Pathologic stage		
IA	28	41.8
IB	20	29.9
IIA	4	6.0
IIB	10	14.9
IIIA	5	7.4

*CT* computed tomography, *FEV*_*1*_ forced expiratory volume in 1 s, *NCD* National Clinical Database, *PET* positron emission tomography, *RC* Risk Calculator, *SUV* standardized uptake value, *VC* vital capacity

^a^The subjects were 59 patients who underwent PET-CT

Their specimens included 33 adenocarcinomas, 23 squamous cell carcinomas, and 11 other types. Mean pathologic tumor size was 32.6 mm (range 13–95 mm). Visceral pleural invasion was observed in 26 patients (38.8%). Lymphatic vessel invasion (LVI) and blood vessel invasion (BVI) were observed in 12 patients (22.6%) and 26 patients (52.0%), respectively. Lymph-node involvement was observed in 8 patients (11.9%).

### Survival analysis

**Fig. 1:**
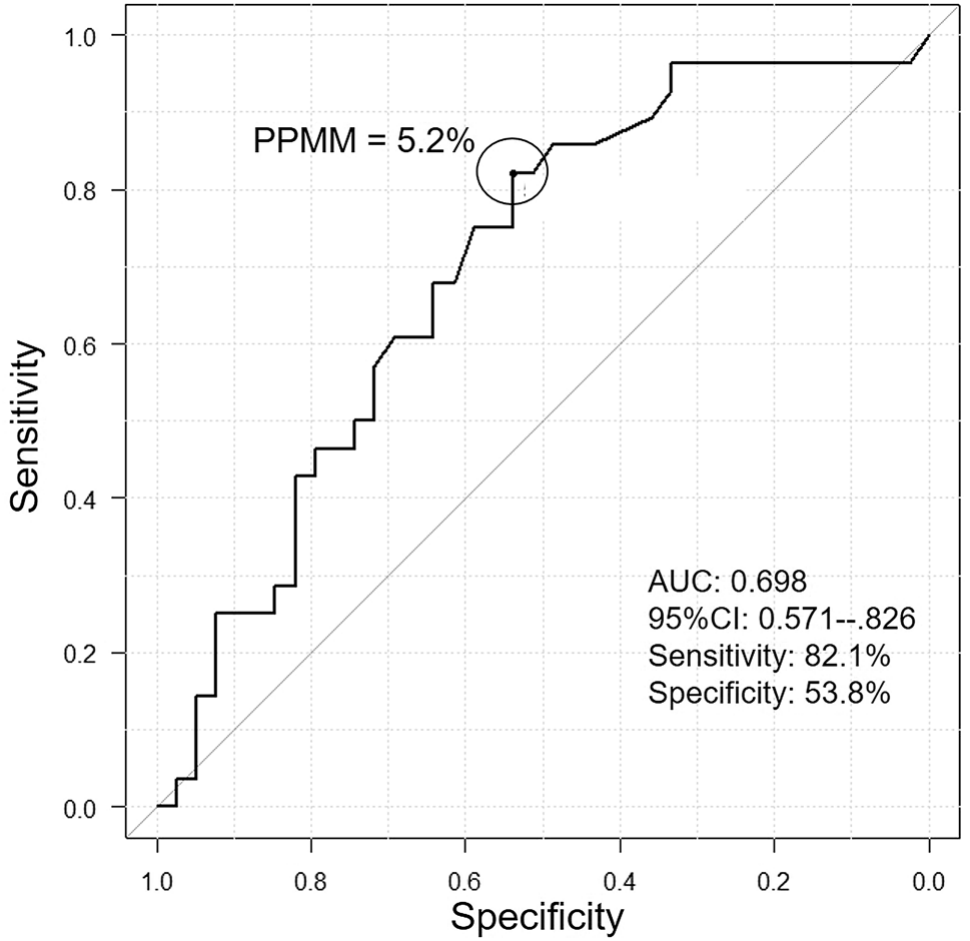
Receiver-operating characteristics curve reveals that the optimal cut-off value for predicting overall survival is predictive probability of perioperative mortality or major morbidity (PPMM) of 5.2% (area under the curve [AUC], 0.698; sensitivity, 82.1%; specificity, 53.8%). *CI* confidence interval

Median follow-up time was 47 months (range 2–102 months). Postoperative local recurrences and distant metastases were observed in 17 patients (25.3%). Eleven patients (16.4%) died from lung cancer. Nine patients (13.4%) suffered sole local postoperative recurrence (Table 2). Five patients with pathological stage I disease had local recurrences. Three patients who had local occurrences underwent additional surgeries, including completion lobectomy or pneumonectomy. The ROC curve identified the optimal PPMM cut-off value of 5.2% (area under the curve [AUC], 0.698; sensitivity, 82.1%; specificity, 53.8%) for predicting overall survival (Fig. 1). Results of univariate and multivariate analyses for each clinicopathological characteristic are listed in Table 3. Significant independent predictive factors related to recurrences were not revealed (Table 3).

**Table 2:** Details of 9 patients who suffered sole local postoperative recurrences

No	Age Sex	Resected segments	ND	Histological type	Pathological tumor size (mm)	LVI BVI	p-Stage	Recurrence site	Treatment to recurrence	Prognosis	Survival after surgery (months)	PPMM with RC (%)
1	81	Lt. S1 + 2, S3	1a	PI	90	ly ( +)	IIB	Lt. S6	None	Dead	20	9.8
	M					v ( +)						
2	81	Lt. S6	1a	Ad	20	ly (−)	IA	Lt. lower lobe	None	Alive	33	11.0
	M					v ( +)						
3	78	Lt. S1 + 2, S3	1b	Sq	35	ly NA	IB	Bronchial stump	Surgery	Alive	100	5.4
	M					v NA			LPN			
4	54	Rt. S6, S10a	2a-1	Ad	21	ly (−)	IA	Resection stump	Surgery	Alive	75	1.4
	F					v (−)			RLL			
5	67	Lt. S1 + 2	1a	Ad	75	ly ( +)	IIB	Resection stump	Surgery	Alive	60	6.5
	M					v ( +)			LUL			
6	86	Rt. S7, S8	1a	PI	27	ly ( +)	IIB	Pleura	None	Dead	6	18.9
	M					v ( +)		Med + hilar LN				
7	77	Rt. S1, S2	1a	Sq	31	ly NA	IB	Resection stump	Surgery	Dead	19	9.4
	M					v NA			RUL			
8	60	Rt. S1, S2	2a-1	AdSq	22	ly NA	IB	Resection stump	CRT	Alive	47	3.5
	M					v NA		Med LN				
9	78 M	Lt. S1 + 2, S3	1b	Ad	32	ly NA	IIA	Resection stump	None	Dead	44	9.6
						v NA		Med + hilar LN				

*Ad* adenocarcinoma; AdSq: adenosquamous carcinoma, *BVI* blood vessel invasion, *CRT* chemoradiation therapy, *LN* lymph node, *LPN* left pneumonectomy, *LUL* left upper lobectomy, *LVI* lymphatic vessel invasion, *Med* mediastinal, *NA* not available, *ND* nodal dissection, *Pl* pleomorphic carcinoma, *PPMM* predictive probability of perioperative mortality or major morbidity, *RC* Risk Calculator, *RLL* right lower lobectomy, *RUL* right upper lobectomy, *Sq* squamous cell carcinoma

**Table 3:** Univariate and multivariate analyses factors related to recurrences in this study cohort

	Univariate analysis		Multivariate analysis	
	OR (95% CI)	*p* value	OR (95% CI)	*p* value
Brinkman Index (> 1000 vs ≤ 1000)	3.000 (0.859–10.476)	0.167		
Tumor size on CT (> 30 mm vs ≤ 30 mm)	1.125 (0.373–3.386)	0.834		
Consolidation/tumor ratio (> 0.75 vs ≤ 0.75)	0.750 (0.248–2.271)	0.259		
SUV of PET-CT (> 5 vs ≤ 5)	0.903 (0.260–3.138)	0.873		
Surgical procedure (complex vs simple segmentectomy)	2.217 (0.692–7.097)	0.180		
Lymph-node dissection (ND0 or ND1 vs ND2)	0.750 (0.248–2.271)	0.611		
Pathologic tumor size (> 30 mm vs ≤ 30 mm)	2.331 (0.759–7.159)	0.139		
Histology (adenocarcinoma vs others)	0.889 (0.295–2.676)	0.834		
Visceral pleural invasion (positive vs negative)	2.184 (0.714–6.678)	0.171		
Lymphatic vessel invasion (positive vs negative)	6.000 (1.425–25.267)	0.0146	1.763 (0.425–7.321)	0.435
Blood vessel invasion (positive vs negative)	14.375 (1.670–123.706)	0.0152	2.146 (0.634–7.269)	0.220
Pathologic lymph-node involvement (N1 or N2 vs N0)	2.464 (0.492–12.354)	0.273		
Pathologic stage (IB-IIIA vs IA)	2.044 (0.627–6.669)	0.217		
PPMM with NCD RC (≥ 5.2% vs < 5.2%)	3.975 (1.015–15.572)	0.0476	3.149 (0.777–12.877)	0.110

*CI* confidence interval, *CT* computed tomography, *NCD* National Clinical Database, *ND* nodal dissection, *OR* odds ratio, *PET* positron emission tomography, *PPMM* predictive probability of perioperative mortality or major morbidity, *RC* Risk Calculator, *SUV* standardized uptake value

**Fig. 2:**
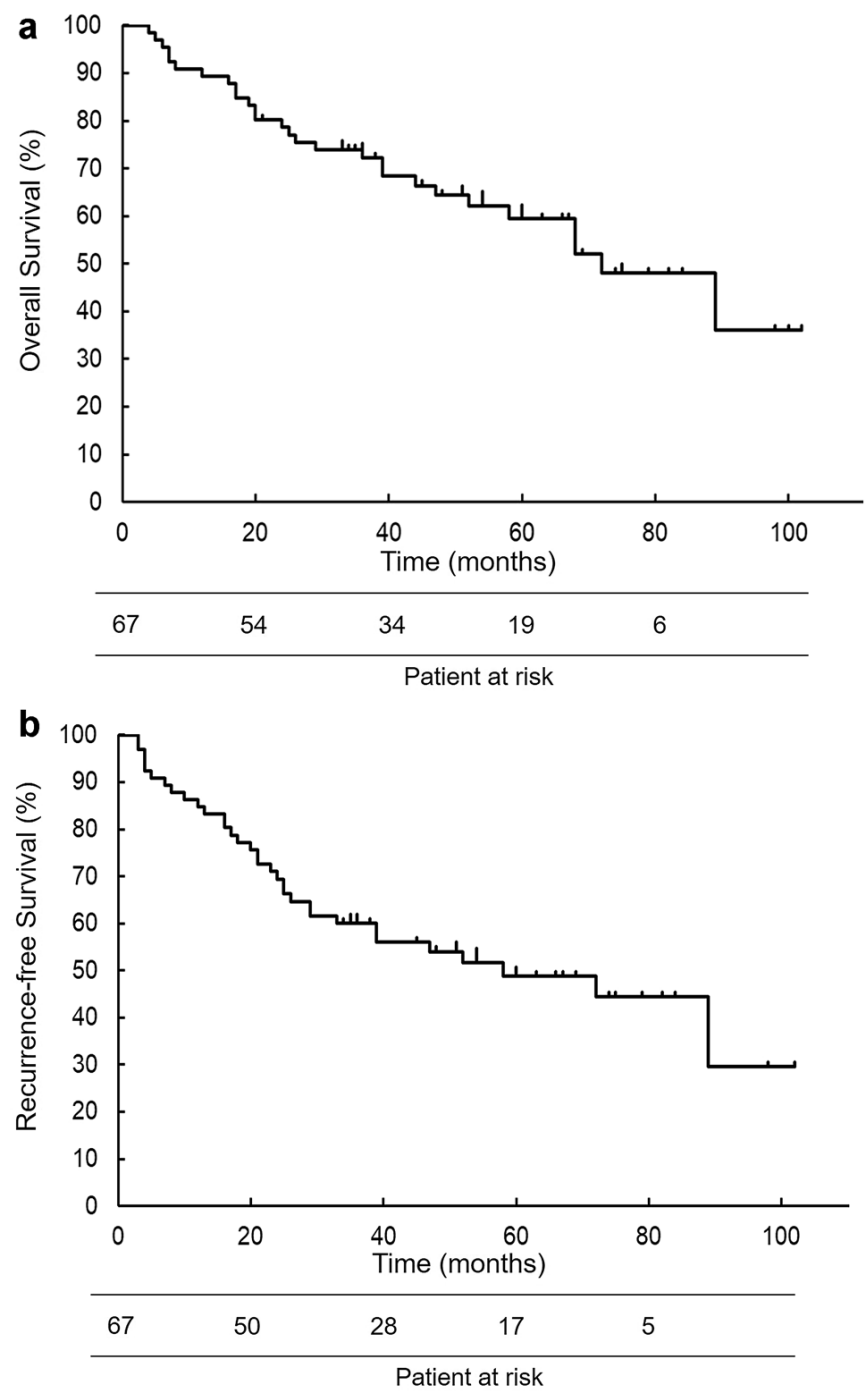
**a** Kaplan–Meier overall survival curve of all patients. **b** Kaplan–Meier recurrence-free survival curve of all patients

Five-year OS and RFS in this cohort were 59.4% and 48.7% (Fig. 2a, b), and did not significantly differ between the groups with pathologic stage IA (OS: 68.4%) disease and pathologic stage IB-IIIA (OS: 52.6%) disease (*P* = 0.37 [OS]; Supplementary Fig. 1). Ten patients (14.9%) died from respiratory diseases other than lung cancer, six (9.0%) from neoplasms of other organs, and cause of death in one patient (1.5%) was unclear.

**Fig. 3:**
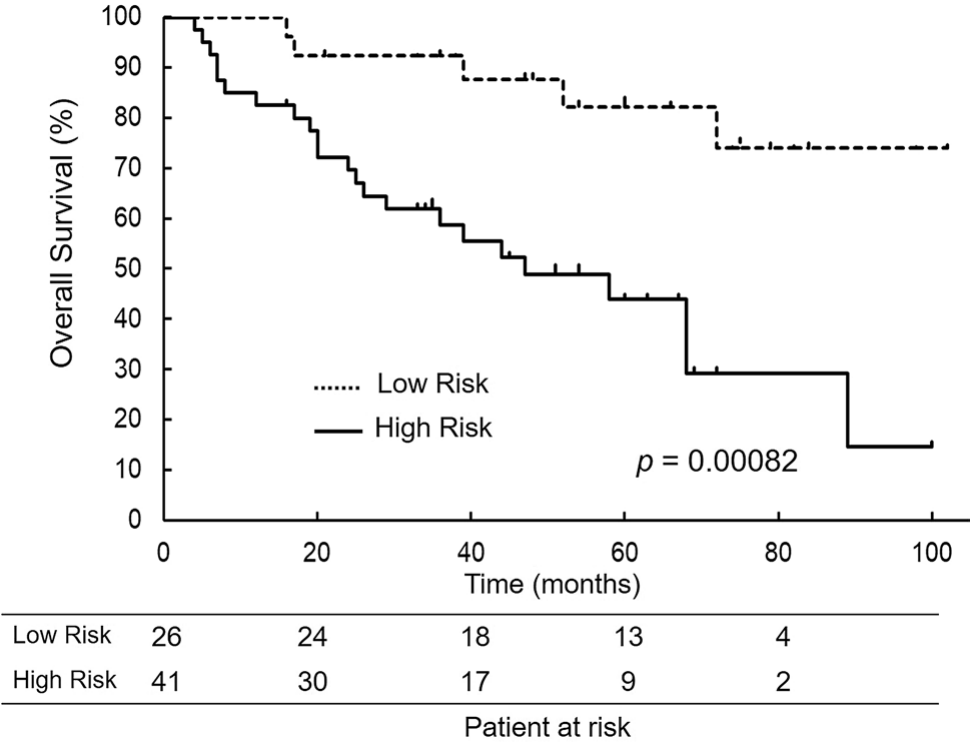
Kaplan–Meier overall survival curves according to predictive probability of perioperative mortality or major morbidity (PPMM) rate

We divided patients by their PPMM into the high-risk (HR) group (PPMM ≥ 5.2%; *n* = 41) and the low-risk (LR) group (PPMM < 5.2%; *n* = 26) in accordance to cut-off value identified by the ROC curve. The HR group had significantly worse 5-year OS (43.9%) than the LR group (82.2%; *P* = 0.00082; Fig. 3). The Cox proportional hazard analysis for the OS demonstrated that a PPMM rate of ≥ 5.2% was the independent predictor (*P* = 0.0116) (Table 4).

**Table 4:** Cox proportional hazard analysis for overall survival in this study cohort

	Univariate analysis		Multivariate analysis	
	HR (95% CI)	*p* value	HR (95% CI)	*p* value
Brinkman Index (> 1000 vs ≤ 1000)	1.526 (0.700–3.327)	0.288		
Tumor size on CT (> 30 mm vs ≤ 30 mm)	2.267 (1.024–5.017)	0.0435	1.523 (0.667–3.477)	0.318
Consolidation/tumor ratio (> 0.75 vs ≤ 0.75)	1.325 (0.617–2.842)	0.471		
SUV of PET-CT (> 5 vs ≤ 5)	2.085 (0.811–5.362)	0.127		
Surgical procedure (complex vs simple segmentectomy)	0.564 (0.228–1.395)	0.215		
Lymph-node dissection (ND0 or ND1 vs ND2)	1.350 (0.628–2.899)	0.442		
Pathologic tumor size (> 30 mm vs ≤ 30 mm)	1.397 (0.665–2.938)	0.378		
Histology (adenocarcinoma vs others)	0.541 (0.251–1.165)	0.116		
Visceral pleural invasion (positive vs negative)	1.667 (0.771–3.605)	0.193		
Lymphatic vessel invasion (positive vs negative)	1.444 (0.517–4.030)	0.483		
Blood vessel invasion (positive vs negative)	3.238 (1.184–8.857)	0.0221	1.347 (0.607–2.988)	0.463
Pathologic lymph-node involvement (N1 or N2 vs N0)	1.849 (0.747–4.580)	0.184		
Pathologic stage (IB-IIIA vs IA)	1.420 (0.654–3.086)	0.376		
PPMM with NCD RC (≥ 5.2% vs < 5.2%)	4.590 (1.724–12.222)	0.00229	3.751 (1.343–10.471)	0.0116

*CI* confidence interval, *CT* computed tomography, *NCD* National Clinical Database, *ND* nodal dissection, *OR* odds ratio, *PET* positron emission tomography, *PPMM* predictive probability of perioperative mortality or major morbidity, *RC* Risk Calculator, *SUV* standardized uptake value

## Discussion

In recent years, several studies have prospectively evaluated segmentectomy versus lobectomy, including multi-institutional randomized clinical trials that address the role of segmentectomy in NSCLC. One is the National Cancer Institute’s Cancer and Leukemia Group B (CALGB) 140,503 trial [12]; another is the JCOG and West Japan Oncology Group trial [9]. Both of these trials evaluate lesions ≤ 2 cm in diameter, as assessed on preoperative CT imaging. However, the present study focuses on PS for patients of advanced age, poor pulmonary function, and/or multiple pulmonary lesions, whose comorbidities could contribute to an unfavorable prognosis after surgery, with lesions ≥ 2 cm in diameter and C/T ratio > 0.5, as assessed on preoperative CT imaging.

The first goal of the present study was to clarify the feasibility of PS for poor-risk patients with NSCLC. In our study cohort, 5-year OS after surgery was 59.4% for all patients, 68.4% for the p-Stage IA group, and 52.6% for the p-Stage IB-IIIA group. In a similar study, estimated 5-year OS for patients who received segmentectomy for node-negative lung cancers, 2–5 cm in size, was 39% [13]. Stereotactic body radiotherapy (SBRT) is often considered as an alternative treatment to palliative limited surgery. In a 2014 study of SBRT in patients aged ≥ 75 years with cT1-2N0M0 NSCLC, 3-year and 5-year OS rates were 73.7% and 43.8%, respectively [14]. In 2018, Timmerman et al. in NRG Oncology Radiation Therapy Oncology Group reported that the 4-year OS of SBRT for operable stage I NSCLC was 56.0%, and the 5-year OS of SBRT for medically inoperable stage I NSCLC was 40.0% [15, 16]. In the context of these reports of SBRT outcomes, outcomes of PS in this study seem quite acceptable. However, 13.4% of patients who underwent PS in this study suffered sole local postoperative recurrences. The variables that significantly related to local recurrences and distant metastases among these patients did not exist in multivariate analysis, and therefore, it is difficult to precisely assess before surgery. PS is an option for poor-risk patients with NSCLC, but its surgical indication should be carefully considered.

The second goal of this study was to clarify predictive factors for prognosis of poor-risk patients with NSCLC. Prognosis of patients in this study did not significantly correlate with pathological lung cancer stage. Multivariate analysis demonstrated that RC (PPMM rate of ≥ 5.2%) was the independent predictor of all-cause mortality. This may be because the patients in this study had poor pulmonary function and other serious diseases, and therefore died from causes other than lung cancer. However, we found that the NCD RC significantly correlated with prognosis in both the perioperative period and over the long term in this study. Risk ratios for postoperative complications are affected by patient demographics, and oncologic factors such as histology and staging, type of surgical procedure, and surgical skill. The STS [8], the US National Cancer Database [17], the European Society of Thoracic Surgeons (ESTS) [18], and institutions in other countries [19] have developed risk models for lung cancer surgery, to assess quality measures for surgeon performance and preoperative decision-making. The results of the present study indicate that RC can be a predictor of long-term prognosis for poor-risk patients who undergo PS for NSCLC. Additionally, our data indicate that selecting SLR over lobectomy cuts the PPMM odds by 2.9% (mean; Table 1). However, an exploration of PPMM from the RC is premature, because the optimal cut-off value to decide between standard surgery and limited surgery remains unclear. The correlation between PPMM from the RC and long-term prognosis should be reviewed with broader data in a future study. The numerical value of the RC PPMM can potentially help to decide surgical suitability and choice of procedure for poor-risk patient.

This study had several limitations. First, this is a two-institution, retrospective study with a small study cohort, which reflects potential selection bias. Second, it did not analyze the role of surgical margins, which might affect local recurrence. The decision to perform limited surgery was each surgeon’s decision without strict criteria, and operative procedures subtly differed with each surgeon. Finally, the NCD and RC database and risk model are insufficiently broad. Endo et al. [6] presented several problematic points regarding the NCD and RC. Although morbidity is defined in the manual for the case report form in the NCD registration system, it is subject to entry error and under-reporting. Input items, including variables and postoperative complications, are handled differently in the NCD than in the STS and ESTS databases; thus, risk models, which are related to regional differences in data collection, should be carefully reviewed. A worldwide clinical database, with the same variables included in all countries, is desirable.

## Conclusions

PS is an option for poor-risk patients with NSCLC, but its surgical indication should be carefully considered because of the higher risks of local recurrence or death by other diseases. The RC, which was primarily developed to determine perioperative risk, can help to determine the appropriateness of surgery and the extent of resection (lobectomy or segmentectomy) for these patients, and predict long-term prognosis for patients who undergo PS.

## Supplementary Information

Below is the link to the electronic supplementary material.

Supplementary file1 (DOC 43 KB)

## References

[1] Ginsberg RJ, Rubinsterin LV (1995). Randomized trial of lobectomy versus limited resection for T1 N0 non-small cell lung cancer Lung Cancer Study Group. Ann Thorac Surg.

[2] Fernando HC, Landreneau RJ, Mandrekar SJ, Nichols FC, Hillman SL, Heron DE (2014). Impact of brachytherapy on local recurrence rates after sublobar resection: results from ACOSOG Z4032 (Alliance), a phase III randomized trial for high-risk operable non–small-cell lung cancer. J Clin Oncol.

[3] Brunelli A, Kim AW, Berger KI, Addrizzo-Harris DJ (2013). Physiologic evaluation of the patient with lung cancer being considered for resectional surgery: diagnosis and management of lung cancer, 3rd ed: American College of Chest physicians evidence-based clinical practice guidelines. Chest.

[4] Miyata H, Gotoh M, Hashimoto H, Motomura N, Murakami A, Tomotaki A (2014). Challenges and prospects of a clinical database linked to the board certification system. Surg Today.

[5] Endo S, Ikeda N, Kondo T, Nakajima J, Kondo H, Yokoi K (2016). Development of an annually updated Japanese national clinical database for chest surgery in 2014. Gen Thorac Cardiovasc Surg.

[6] Endo S, Ikeda N, Kondo T, Nakajima J, Kondo H, Yokoi K (2017). Model of lung cancer surgery risk derived from a Japanese nationwide web-based database of 78 594 patients during 2014–2015. Eur J Cardiothorac Surg.

[7] Kozower BD, Sheng S, O’Brien SM, Liptay MJ, Lau CL, Jones DR (2010). STS database risk models: predictors of mortality and major morbidity for lung cancer resection. Ann Thorac Surg.

[8] Fernandez FG, Kosinski AS, Burfeind W, Park B, DeCamp MM, Seder C (2016). STS lung cancer resection risk model: higher quality data and superior outcomes. Ann Thorac Surg.

[9] Nakamura K, Saji H, Nakajima R, Okada M, Asamura H, Shibata T (2010). A phase III randomized trial of lobectomy versus limited resection for small-sized peripheral non-small cell lung cancer (JCOG0802/WJOG4607L). Jpn J Clin Oncol.

[10] Aokage K, Saji H, Suzuki K, Mizutani T, Katayama H, Shibata T (2017). A non-randomized confirmation trial of segmentectomy for clinical T1N0 lung cancer with dominant ground glass opacity based on thin-section computed tomography (JCOG1211). Gen Thorac Cardiovasc Surg.

[11] The Japanese Lung Cancer Society (2010). General rule for clinical and pathological records of lung cancer.

[12] Altorki NK, Wang X, Wigle D, Gu L, Darling G, Ashrafi AS (2018). Perioperative mortality and morbidity after sublobar versus lobar resection for early-stage non-small-cell lung cancer: post-hoc analysis of an International, randomised, phase 3 Trial (CALGB/Alliance 140503). Lancet Respir Med.

[13] Stiles BM, Mao J, Harrison S, Lee B, Port JL, Altorki NK (2019). Sublobar resection for node-negative lung cancer 2–5 cm in size. Eur J Cardiothorac Surg.

[14] Nakagawa T, Negro Y, Matsuoka T, Okumura N, Dodo Y (2014). Comparison of the outcomes of stereotactic body radiotherapy and surgery in elderly patients with cT1-2N0M0 non-small cell lung cancer. Respir Investig.

[15] Timmerman RD, Paulus R, Pass HI, Gore EM, Edelman MJ, Galvin J (2018). Stereotactic body radiation therapy for operable early-stage lung cancer: Findings from the NRG Oncology RTOG 0618 trial. JAMA Oncol.

[16] Timmerman RD, Hu C, Michalski JM, Bradley JC, Galvin J, Johnstone DW (2018). Long-term results of stereotactic body radiation therapy in medically inoperable stage I non-small cell lung cancer. JAMA Oncol.

[17] Khullar OV, Gillespie T, Nickleach DC, Liu Y, Higgins K, Ramalingam S (2015). Socioeconomic risk factors for long-term mortality after pulmonary resection for lung cancer: an analysis of more than 90,000 patients from the National Cancer Data Base. J Am Coll Surg.

[18] Brunelli A, Salati M, Rocco G, Varela G, Van Raemdonck D, Decaluwe H, ESTS Database Committee (2016). European risk models of for morbidity (EuroLung1) and mortality (EuroLung2) to predict outcome following anatomic lung resections: an analysis from the European Society of Thoracic Surgeons database. Eur J Cardiothorac Surg.

[19] Falcoz PE, Puyraveau M, Rivera C, Bernard A, Massard G, Mauny F (2014). The impact of hospital and surgeon volume on the 30-day mortality of lung cancer surgery: a nation-based reappraisal. J Thorac Cardiovasc Surg.

