# Supplementary material for: Can the Japanese National Clinical Database risk calculator predict long-term survival of patients who undergo palliative segmentectomy for primary lung cancer?
Source: Gen Thorac Cardiovasc Surg. 2021 Jan 28;69(7):1096–104. doi: 10.1007/s11748-021-01585-6 (PMC8203513; doi:10.1007/s11748-021-01585-6)
Supplement: Supplementary file 1 — Supplementary file1 (DOC 43 KB) [file 11748_2021_1585_MOESM1_ESM.doc]

**Supplementary Table 1 Predictors of mortality and composite mortality/major morbidity.**

|  | *P-value* | *OR (95% CI)* |
| --- | --- | --- |
| Mortality model  Male  Five-year increase in age (60-79 years)  PS  PS1  PS2 or higher  %VC 10% decrease (from 100% to 50%)  Liver cirrhosis (Child-Pugh Class B/C)  Hemodialysis  Interstitial pneumonia  Ischemic heart disease (with/without intervention)  Smoking history  Tumor size > 3cm (radiological)  Clinical stage ^a^  II or higher  III or higher  Superior sulcus tumor  Surgical procedure  Right lower lobectomy  Lobectomy or bilobectomy  Pneumonectomy  Chest wall resection (other than first rib)  Histology other than adenocarcinoma  Mortality and morbidity model  Male  Five-year increase in age (60-79 years)  Cigarette smoking 30 pack-year or more  PS  PS1  PS2 or higher  %VC 10% decrease (from 100% to 50%)  %FEV_1_ < 70%  %FEV_1_ < 50%  Hemodialysis  Interstitial pneumonia  Stroke  Untreated diabetes mellitus  Autoimmune disease  Arrhythmia  Induction radiotherapy or chemoradiotherapy  Clinical stage II or higher  Surgical procedure  Pneumonectomy  Lobectomy or bilobectomy  Nodal dissection  Hilar or lobe specific or systematic  Systematic  Combined resection  Pulmonary artery  Chest wall (other than first rib)  Chest wall (first rib)  Wedge resection or segmentectomy of lung  Histology other than adenocarcinoma | <0.001  <0.001  0.006  <0.001  <0.001  0.009  0.006  <0.001  0.023  0.019  0.027  0.006  0.009  0.032  0.001  <0.001  <0.001  <0.001  0.001  <0.001  <0.001  <0.001  0.001  <0.001  <0.001  0.002  <0.001  0.003  <0.001  0.040  0.021  0.016  <0.001  <0.001  <0.001  <0.001  <0.001  <0.001  <0.001  0.019  0.005  0.004  <0.001  <0.001 | 2.366 (1.533-3.651)  1.420 (1.299-1.551)  1.457 (1.113-1.908)  2.644 (1.836-3.806)  1.380 (1.277-1.491)  3.075 (1.320-7.161)  2.883 (1.357-6.125)  3.690 (2.790-4.880)  1.504 (1.057-2.140)  1.711 (1.093-2.677)  1.354 (1.036-1.771)  1.537 (1.130-2.091)  1.568 (1.120-2.196)  1.752 (1.048-2.931)  1.604 (1.213-2.122)  1.973 (1.382-2.816)  5.224 (2.865-9.523)  2.820 (1.584-5.019)  1.502 (1.181-1.911)  1.724 (1.519-1.917)  1.160 (1.124-1.197)  1.236 (1.105-1.382)  1.228 (1.094-1.379)  1.473 (1.216-1.784)  1.148 (1.108-1.188)  1.164 (1.055-1.284)  1.506 (1.213-1.870)  1.847 (1.226-2.781)  2.293 (1.978-2.658)  1.182 (1.007-1.387)  1.567 (1.069-2.299)  1.405 (1.065-1.854)  1.849 (1.554-2.201)  1.762 (1.347-2.304)  1.341 (1.209-1.487)  3.092 (2.296-4.165)  1.475 (1.248-1.743)  1.999 (1.621-2.465)  1.210 (1.096-1.335)  1.721 (1.095-2.706)  1.592 (1.152-2.199)  2.584 (1.356-4.925)  1.558 (1.222-1.986)  1.229 (1.115-1.354) |

*CI* confidence interval, *FEV_1_* forced expiratory volume in 1 second, *OR* odds ratio, *PS* performance status, *VC* vital capacity

^a^Clinical stage 7^th^ edition TNM classification by UICC
